# Root Hair Single Cell Type Specific Profiles of Gene Expression and Alternative Polyadenylation Under Cadmium Stress

**DOI:** 10.3389/fpls.2019.00589

**Published:** 2019-05-10

**Authors:** Jingyi Cao, Congting Ye, Guijie Hao, Carole Dabney-Smith, Arthur G. Hunt, Qingshun Q. Li

**Affiliations:** ^1^Department of Biology, Miami University, Oxford, OH, United States; ^2^Cell, Molecular, Structural Biology Graduate Program, Miami University, Oxford, OH, United States; ^3^Key Laboratory of Ministry of Education for Coastal and Wetland Ecosystems, College of the Environment and Ecology, Xiamen University, Xiamen, China; ^4^Department of Plant and Soil Sciences, University of Kentucky, Lexington, KY, United States; ^5^Department of Chemistry and Biochemistry, Miami University, Oxford, OH, United States; ^6^Graduate College of Biomedical Sciences, Western University of Health Sciences, Pomona, CA, United States

**Keywords:** Arabidopsis, cell-type specific, mRNA processing, alternative polyadenylation, PAT-Seq, root hair, cadmium stress

## Abstract

Transcriptional networks are tightly controlled in plant development and stress responses. Alternative polyadenylation (APA) has been found to regulate gene expression under abiotic stress by increasing the heterogeneity at mRNA 3′-ends. Heavy metals like cadmium pollute water and soil due to mining and industry applications. Understanding how plants cope with heavy metal stress remains an interesting question. The Arabidopsis root hair was chosen as a single cell model to investigate the functional role of APA in cadmium stress response. Primary root growth inhibition and defective root hair morphotypes were observed. Poly(A) tag (PAT) libraries from single cell types, i.e., root hair cells, non-hair epidermal cells, and whole root tip under cadmium stress were prepared and sequenced. Interestingly, a root hair cell type-specific gene expression under short term cadmium exposure, but not related to the prolonged treatment, was detected. Differentially expressed poly(A) sites were identified, which largely contributed to altered gene expression, and enriched in pentose and glucuronate interconversion pathways as well as phenylpropanoid biosynthesis pathways. Numerous genes with poly(A) site switching were found, particularly for functions in cell wall modification, root epidermal differentiation, and root hair tip growth. Our findings suggest that APA plays a functional role as a potential stress modulator in root hair cells under cadmium treatment.

## Introduction

Heavy metals are naturally occurring components in soil. However, increases in industrial manufacturing in the last two centuries have led to significant increases of heavy metal pollution in the environment. Plants grown under elevated heavy metal conditions show inhibited growth and reduced yield (Chibuike and Obiora, 2014). One of these metals, cadmium (Cd), is a non-essential element in plant growth and is highly toxic to plants (Tran and Popova, 2013). Cd is released into soil though the use of fertilizers as well as various industrial processes such as additives in plastic stabilizers, NiCd batteries, pigments, and electroplating (Sanitá di Toppi and Gabbrielli, 1999). Besides the effects of Cd on plant growth and development, it has been reported that Cd can accumulate in plant products like rice grains, leading to loss of production and contamination of the food chain (Chunhabundit, 2016). How plants cope with Cd-polluted soil environments remains to be well understood.

A majority of plant species (excluder plants) have evolved strategies to restrict heavy metal translocation and retain low amounts of metal contaminants in aerial tissues (Baker, 1981). For example, in Arabidopsis roots are the major organ for metal accumulation. The cell wall, containing polysaccharides, functions as the first barrier of heavy metal accumulation by blocking heavy metals from entering the cytosol (Parrotta et al., 2015). Excessive amounts of heavy metals are further immobilized by phytochelatins (PCs) and sequestered to vacuoles (Hall, 2002). The negative effects of Cd have been observed through reductions in photosynthesis rate and root elongation, and have been suggested to be a generic stress-induced morphogenic response (SIMR) similar to those detected with other heavy metals or UV-B radiation (Parrotta et al., 2015). A signature of SIMR, e.g., clustered root hairs near the apex, was shown in rapeseed under Cd stress (Sun and Guo, 2013). However, no molecular mechanism has been suggested to explain this phenomenon.

One aspect in post-transcriptional regulation, alternative splicing, has been shown to alter expression of specific genes though intron retention in fungi under Cd stress (Georg et al., 2009). Additionally, a variety of alternative splicing events including exon skipping, intron retention, and alternative usage of 5′ and 3′ splice sites were identified in rice under Cd stress (He et al., 2015). Another aspect in post-transcriptional regulation, alternative polyadenylation (APA), has been documented to regulate gene expression in abiotic stress responses (Zhang et al., 2008). Together with alternative splicing, APA plays a functional role in drought stress in sorghum (Abdel-Ghany et al., 2016). Furthermore, APA was revealed as a common phenomenon in eukaryotes for other biological processes including embryonic development and cell differentiation (Tian and Manley, 2017). Previous studies found the usage of multiple polyadenylation sites in tissue- and developmental-specific APA in zebrafish (Ulitsky et al., 2012), worms (Mangone et al., 2010), and rice (Fu et al., 2016). More recently, one study on plant hypoxia stress investigated the regulatory function of APA through production of non-canonical mRNA isoforms (De Lorenzo et al., 2017).

Root hairs function in absorption and perception, but they are also an attractive cellular model in both molecular and physiological studies due to their single cell properties as simple structures and tubular outgrowth of hair-forming root epidermis (Qiao and Libault, 2013). An RNA-Seq survey of Arabidopsis root hair cells showed a substantial number of alternative splicing events enriched in biological processes linked to Cd stress (Lan et al., 2013). However, the underlying mechanism of Cd-induced root hair morphotypes is still largely unknown.

A map of cell type-specific alternative splicing was revealed in Arabidopsis root by combined fluorescence-activated cell sorting (FACS) and next generation sequencing (Li et al., 2016). Application of the same methodology using FACS and microarray identified the core regulators in abiotic stress responses (Dinneny et al., 2008). Construction of two cell type markers (root hair, and non-hair epidermal cells) in Arabidopsis using a system of isolating nuclei tagged in specific cell-types (INTACT) allowed the transcriptome profiling of Arabidopsis root hairs (Deal and Henikoff, 2011). Herein, we applied FACS and Illumina sequencing to explore the regulatory function of APA in plant root hair development under Cd stress.

## Materials and Methods

### Plant Material

*Arabidopsis thaliana* (L.) Heynh. ecotype Col-0 (CS60000) was used in this study. Two GFP reporter lines, ADF8p:NTF/ACT2p:BirA and GL2p:NTF/ACT2p:BirA]kindly provided by Dr. Roger Deal, now at Emory University, (Deal and Henikoff, 2010)], were used for cell sorting. In the cell line ADF8p:NTF/ACT2p:BirA, GFP was fused with cell type-specific promoter ADF8, which expresses green fluorescence in root hair cells. In the other cell line GL2p:NTF/ACT2p:BirA, the cell type-specific promoter GL2 drives GFP expression only in non-hair root epidermal cells. Seeds were surface sterilized with 50% bleach and 0.05% Tween 20 for 8 min. After rinsing 5 times with sterile distilled water, seeds were stratified at 4°C for 2 days and grown on top of a 100 μm nylon mesh (Genesee Scientific) of ½ Murashige and Skoog (MS) (Sigma-Aldrich) medium with 1% sucrose, 1% agar at 22°C vertically with a 16 h light / 8 h dark cycle. Five-day-old seedlings were transferred to ½ MS medium (control) or ½ MS medium containing 100 μM CdCl_2_ (stress) for 24, 48, and 72 h. Seeds were plated in a density of ∼20 / cm in two rows on top of 100 μm nylon mesh for the sorting experiment in three biological replicates.

### Phenotypic Measurement

Seedlings of wild-type Col-0 were grown on ½ MS agar plates for 5 days and transferred to ½ MS medium (control) or ½ MS medium containing 100 μM CdCl_2_ (stress) for 24, 48, and 72 h for phenotypic analysis. Seedlings were imaged and root length was measured using *Image J* (National Institute of Health; http://rsb.info.nih.gov/ij). In total, 35–40 seedlings were used in each experiment and experiments were repeated four times. Means and standard errors were analyzed by a Repeated Measures ANOVA using SAS (SAS Institute Inc., NC, United States).

### Protoplasting and Fluorescence-Activated Cell Sorting

The specific GFP expression in each marker line was confirmed by confocal microscopy. Briefly, seedlings were stained with 200 μg/ml propidium iodide for 2 min, rinsed with water, and imaged with a Carl Zeiss LSM710 confocal microscope. Protoplasting was performed as described previously (Bargmann and Birnbaum, 2010) with slight modification. Briefly, 1.25% (w/v) cellulase (Calbiochem, #Cat219466) and 0.3% (w/v) macerozyme R-10 (Research Product International, Cat#M22010) were used for root cell protoplasting. Enzymes were dissolved in a washing solution [0.6 M Mannitol, 1 mg/ml BSA, 10 mM MgCl_2_⋅6H_2_O, 10 mM CaCl_2_⋅2H_2_O, 10 mM KCl, 0.39 mg/ml MES hydrate, pH 5.5 adjusted with 1M Tris]. Chopped root tips were incubated at room temperature for 1 h with 85 rpm agitation. Protoplast suspension was centrifuged at 1,200 rpm for 6 min (RT). Protoplast pellets were then washed and filtered through both 70 μm and 40 μm cell strainers (Fisher Scientific).

The fluorescence-activated cell sorter Moflo XDP (Beckman Coulter, Inc) with a 100 μm nozzle (Lan et al., 2013) was used for collecting the GFP-positive cells, at a rate of 2,000 to 5,000 events per second at a fluid pressure of 25 psi (Research Flow Cytometry Core at Cincinnati Children’s Hospital Medical Center). The roots tips of Col-0 were used as negative control to collect GFP-labeled cells and the expected sorting event number is about 5,000 to 20,000 cells.

### RNA Isolation

Collected cells were sorted into RA1 lysis buffer (within the kit for RNA isolation) supplemented with the reducing agent tris 2-carboxyethl phosphine (TCEP) at room temperature. Total RNA was extracted immediately after cell sorting (NucleoSpin RNA XS kit, Macherey Nagel) with an on-column treatment with RNase-free DNase I (provided with the kit) following the manufacturer’s recommendations. Quality and quantity of extracted total RNA were assessed by RNA pico chip using Agilent Bioanalyzer 2100 (Agilent Biotechnologies). Samples with an RNA integrity number (RIN) above 6.9 were stored at -80°C for library construction.

### Preparation for Poly(A) Tag Library

Poly(A) tag libraries were constructed as described (Cao and Li, 2015, Pati et al., 2015) with modifications. Briefly, about 10 ng of total RNA was mixed with 100 pmol of anchored, oligo-dT primer with barcodes (RT-PEXX, final conc. of 6.7 pmol/μL) (Supplementary Table S1) and 5X first strand buffer (supplied with SMARTScribe reverse transcriptase), then incubated at 95°C for 2 min fragmentation followed immediately by chilling on ice. To perform the first strand cDNA synthesis, fragmented RNA and oligonucleotides were incubated at 65°C for 5 min and chilled on ice. This process was repeated once and then mixed with buffer components and dNTPs. One microliter of SMARTScribe reverse transcriptase (Clontech #Cat 639537) was added and incubated at 42°C for 2 h on a preheated PCR block. An additional 1 μL of SMARTSCRIBE reverse transcriptase was added and incubation continued at 42°C for an additional 2 h. Then, 100 pmol (final conc. of 4 pmol/μL) of the locked nucleic acid (LNA; Exiqon Inc) oligo optimized for strand-switching reaction and 1 μL of SMARTSCRIBE were added and incubated in the programmed PCR block at 42°C for 2 h, followed by 70°C for 5 min to inactive the enzyme. cDNA was purified twice by AMPure beads (Beckman Coulter, Inc) and eluted in 20 μl nuclease-free water. A 20-μL reaction was performed using 1.5 μL of cDNA. A total of 4 pmol of PE-PCR 1.0 and PE-PCR 2.0 primers was added for 18–25 cycles of PCR (each cycle consisting of 95°C melting for 2 min, 60°C annealing for 1 min, 72°C extension for 1 min, followed by a polish step at 72°C for 10 min). The smear appearing at 250–400 bp on the agarose gel was purified on a NucleoSpin column (Macherey Nagel). Purified cDNA was used as a template for additional PCR with 4 pmol of P5 and P7 primers for 5–12 cycles. Libraries were examined by a high-sensitivity DNA chip (Agilent Biotechnologies) and quantified by a KAPA library quantification kit (KAPA Biosystems). The final PAT-Seq libraries were submitted for Illumina HiSeq 2500 sequencing (Core Facility of the College of the Environment and Ecology, Xiamen University, Xiamen, Fujian, China). The raw data have been deposited to the NCBI SRA (Accession number: PRJNA477396).

### Data Analysis to Identify Poly(A) Site Usage

A bioinformatics pipeline was developed for data processing and analysis (Wu et al., 2015) with minor modification. Briefly, raw data generated in fastq format were filtered using a FASTX-Toolkit (-q 10 –p 50 –v –Q 33). Using a customized Perl script, the Illumina PE2 sequence and the sequence of poly(T) were removed from PATs. Reads with no oligo-dT were discarded. Retained reads shorter than 20 nt were also discarded. The trimmed PATs were mapped to the Arabidopsis TAIR 10 genome using Bowtie2 (Langmead et al., 2009). The mapped PATs were converted into bed files and then sorted by using Bedtools2 (Quinlan and Hall, 2010). Potential PATs were extracted by using a customized Perl script and annotated by customized MATLAB scripts. To avoid false positives, PATs caused by internal priming were removed (Loke et al., 2005). PATs at the same coordinates were grouped into one poly(A) site (PAS). Poly(A) clusters (PACs) were identified by grouping the PATs within 24 nt into a transcription unit (Wu et al., 2011). PACs supported by less than 10 reads across all samples were discarded. The location of the PAS with the most abundant reads in a cluster was chosen to represent the cluster. The sorted list of PACs were annotated using a GFF file in which all 3′ UTRs were extended by 120 nt (Wu et al., 2011). Alignment information of FACS-sorted samples is listed in Supplmentary Table S2.

For detection of differentially expressed genes, counts collected from PACs located on the same gene were combined and normalized by DEseq2 (Love et al., 2014). Two-sample comparison was performed by DEseq2 for identifying differentially expressed genes [adjusted *p* value (*padj*) < 0.01]. For detection of differentially expressed PACs, DEseq2 was used to normalize the read counts. Two-sample comparison was performed by DEseq2 for identifying significant differentially expressed PAC (*padj* < 0.05). For identification of poly(A) site switching genes, genes with at least two PACs were selected and analyzed (Mangone et al., 2010; Wu et al., 2011). Poly(A) site switching genes were detected by two-sample comparison. Briefly, for genes with more than two PACs, any two PACs were denoted as PA1 and PA2. Then, genes meeting the following criteria were considered as APA switching instances: (1) one of PA1 and PA2 at least has a read count ≥ 5 in each sample; (2) at least one of PA1 and PA2 has total read count across all samples ≥ 20; (3) PA1/PA2 read count ratio ≥ 2 in one sample and PA2/PA1 also ≥ 2 in another sample; (4) read counts of | PA1 - PA2| > 5 within each sample in which switching occurred; and (5) they were also detected with DE-PAC usage. To determine the motif around PAC in specific genomic regions (intergenic, CDS, and 3′ UTR), a customized Perl script was applied to retrieve sequence from -300 to 100 nts around the targeted PACs. Selected sequences were analyzed by MEME (Bailey et al., 2009) for *de novo* motif detection. ClusterProfiler (Yu et al., 2012) was used for GO enrichment analysis (FDR < 0.05). The R package org.At.tair.db was applied for GO term annotation. Venn diagrams were created using EucGenIE^1^ (Bardou et al., 2014).

## Results

### Cadmium Inhibits Root Growth and Development

**FIGURE 1:**
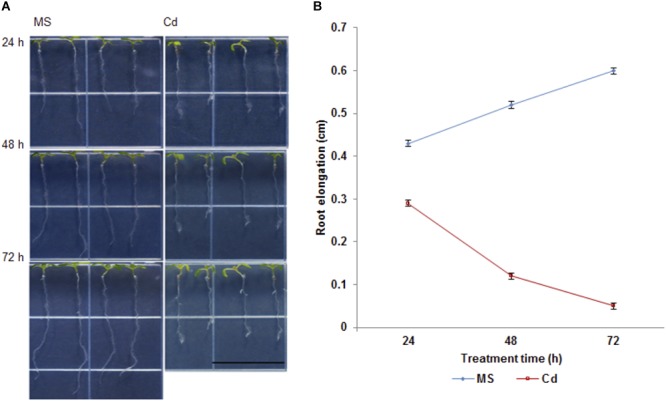
Cadmium affects primary root growth in *Arabidopsis thaliana*. **(A)** Representative seedling growth of Arabidopsis germinated on ½ MS medium for 5 days and then transferred to ½ MS (control) and 100 μM CdCl_2_ for 24, 48, and 72 h. Note that the root hair clusters shift to root apex. Bar = 0.5 cm. **(B)** Net growth of primary root upon cadmium stress.

**FIGURE 2:**
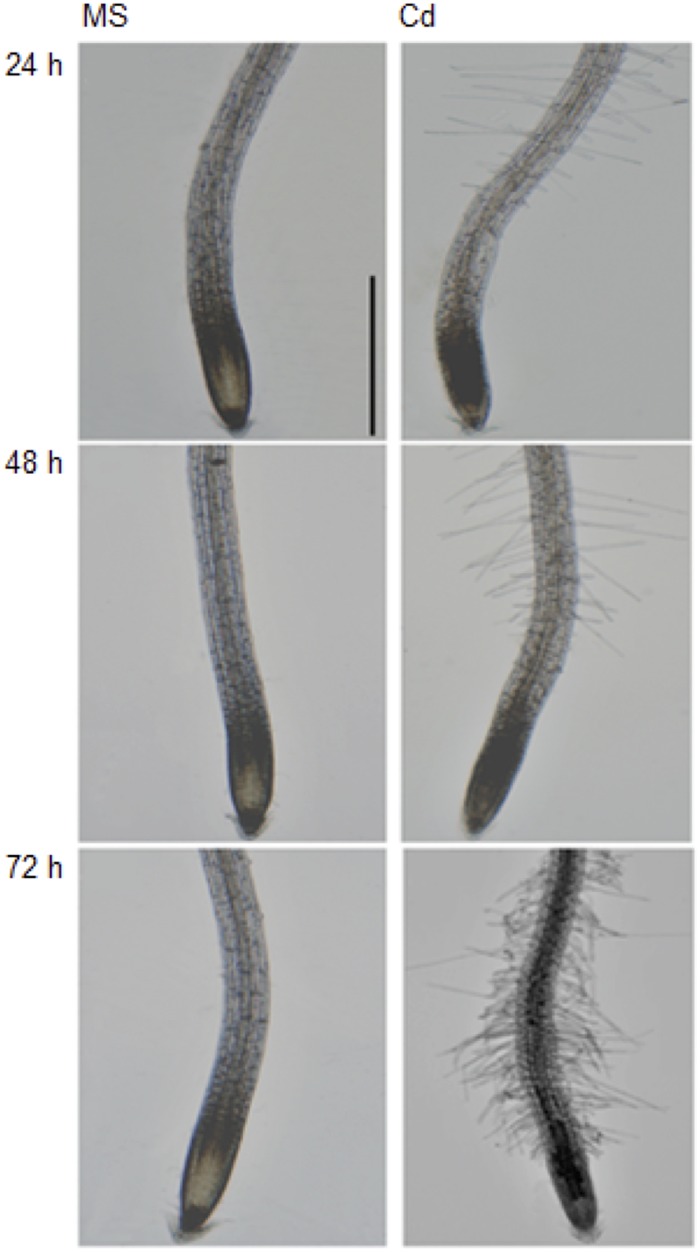
Cadium induces subapical root hair formation and root hair morphotypes. Root tips grown on control medium and Cd stress medium for 24, 48, and 72 h. Scale bar is 100 μm.

It has been documented that Cd (75 to 150 μM CdCl_2_) inhibits plant root growth in a concentration-dependent manner (Yuan and Huang, 2015). In order to further investigate the physiological and morphological effects of Cd on root growth in Arabidopsis, 5-day-old seedlings were transferred to half strength MS medium (control) or ½ MS supplemented with 100 μM CdCl_2_ (stress) and changes were observed in primary root length after 24, 48, and 72 h (Figure 1A). We found that root growth was significantly decreased after 48 h of Cd treatment (Figure 1B) with subtle growth after 72 h. Therefore, Cd repressed root growth in a time-dependent manner. Additionally, migration of the root hair zone toward the root tip was observed (Figure 2). The occurrences of subapical root hairs were dramatically increased as incubation time increased. Deformities in root hair tip growth were detected with either basipetal or a topical enlargement from prolonged Cd stress after 48 h and 72 h (Figure 2).

### Isolation of Arabidopsis Root Hairs and PAT-Seq Library Production

**FIGURE 3:**
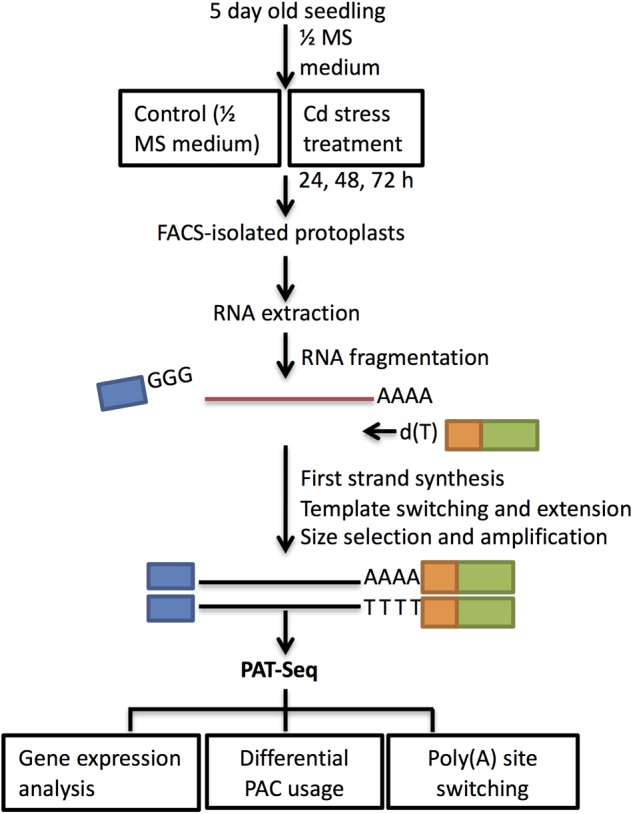
Schematic workflow of cell type isolation and sequencing analysis. Seedlings of wild type Col-0 were grown on ½ MS medium for 5 days and then transferred to ½ MS (control) and 100 μM CdCl_2_ for 24 h, 48 h, and 72 h. Section around ∼1.25 cm between the starting root hair differentiation zone and first lateral root was harvested at each time point for protoplasting and FACS sorting. Total RNA isolated from the collected cells was used for PAT library preparation. First-strand cDNA was primed by oligo d(T)-attached RT-PE primers and used SMART 7.5 for template switching at 5**′** end (Supplementary Table S1). After size selection and PCR amplification, samples were sequenced on Illumina HiSeq 2500. PAT-Seq data were analyzed for differentially expressed genes, PACs, and identification of poly(A) switching genes.

The INTACT technique uses fused GFP expression targeting to the nuclear envelope to facilitate cell sorting and was first applied in Arabidopsis root hair and non-hair epidermal cells (Deal and Henikoff, 2011). Our findings of inhibited root growth, and especially the induced root hair cluster at the root apex, prompted our interest to investigate gene expression changes during Cd response in root hair cells. Cell fate specification of root hair cells is well defined in Arabidopsis, which was determined by the relative abundance of transcription factors due to position-based signals (Lee and Schiefelbein, 1999). As defined, epidermal cells located at the junction of two cortical cells differentiate into root hair cells, and the epidermal cells at the top of the cortical cells develop into non-hair cells (Masucci et al., 1996). In order to minimize the diluting factors from whole-organ analysis, FACS sorting has been widely applied in transcriptomic studies in specific cell types and under stress (Birnbaum et al., 2005). Additionally, the INTACT method was previously established for profiling gene expression and histone modification from Arabidopsis root hairs (Deal and Henikoff, 2010). Here we used two GFP marker lines—ADF8p:NTF/ACT2p:BirA (Supplementary Figure S1A) and GL2p:NTF/ACT2p:BirA (Supplementary Figure S1B)—for collecting root hair cells and non-hair epidermal cells, respectively. The section starting at the root hair zone until the first lateral root was harvested for FACS sorting (Figure 3). Libraries for PAT-Seq were constructed using SMART technology with a typical input of 10 ng total RNA isolated from collected cell populations (Pati et al., 2015).

### The Impact of Cd Stress on Root Hair-Specific Gene Expression Stress

**FIGURE 4:**
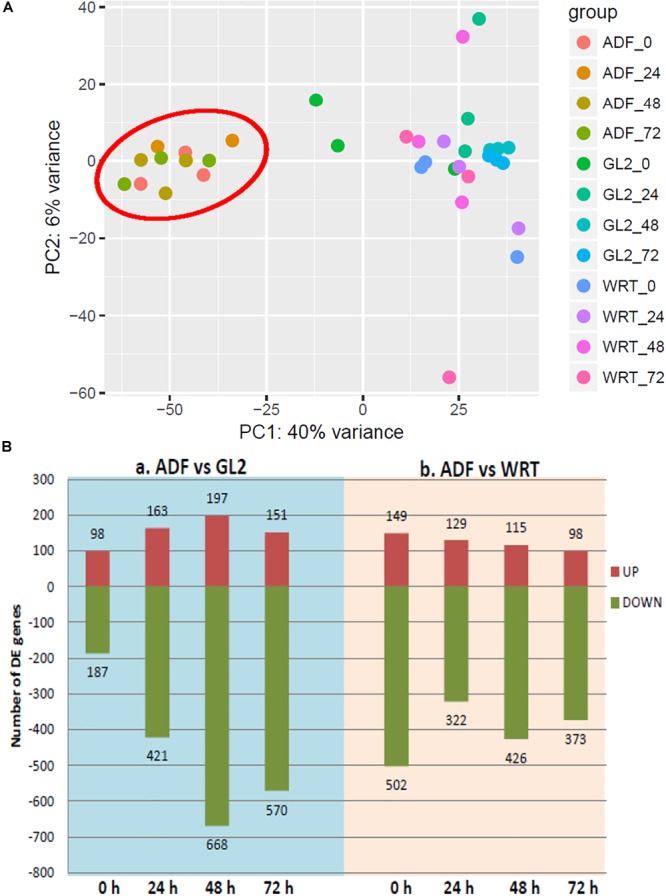
Differentially expressed genes (DE-gene) under cadmium stress. **(A)** Principle component analysis of differential gene expression across each cell type. **(B)** Number of up- and down-regulated DE-genes detected from sample-to-sample comparison. ADF represents root hair cells, GL2 represents non-hair epidermal cells, and WRT represents whole root tip at time point of 0, 24, 48, and 72 h of Cd stress. Root hair-specific cluster is circled in red.

PAT-Seq results provided two sets of transcriptome-related information: the expression level of cell type-specific gene expression under Cd, and the collection of poly(A) site and clusters. The collected poly(A) tags were first pooled for analysis of differential gene expression as the tag number represents the expression level of genes (Wu et al., 2011). A pair-wise sample-to-sample comparison was carried out, and the detected gene expression levels from root hair cells (ADF8p:NTF/ACT2p:BirA) were compared to whole root tip (WRT) control and non-hair epidermal cells (GL2p:NTF/ACT2p:BirA) at multiple time periods of Cd treatment (0, 24, 48, and 72 h) (Figure 4). We found a total of 149 up-regulated genes involved in cell wall organization, root development, and root hair cell differentiation from the comparison of root hair cells to the WRT control of 5-day-old seedlings using PAT-Seq. Most of these genes (122 of 149) were also identified as enriched genes in root hairs from published data sets by tilling array (Deal and Henikoff, 2010), microarray (Becker et al., 2014), and RNA-Seq (Lan et al., 2013) (Supplementary Figure S2). The highest overlapping detection rate (107 out of 149, 72%) was achieved in comparison with the microarray data set (Becker et al., 2014), validating the approach taken in this study.

Upon sequencing of the PAT-Seq libraries, the profile of poly(A) sites was analyzed. We pooled the surrounding regions of PACs for single nucleotide composition analysis of poly(A) signals. Similar to what has been revealed from Arabidopsis leaf and seed tissue (Wu et al., 2011), we found an A+T-rich signal used in 3′ UTR (Supplementary Figure S3A) and an A+G-rich signal used in coding regions (Supplementary Figure S3B). The cell type-specific distribution of PATs and PACs is similar to our earlier studies with normal PAT-Seq of whole tissue samples (Wu et al., 2011; Fu et al., 2016).

**FIGURE 5:**
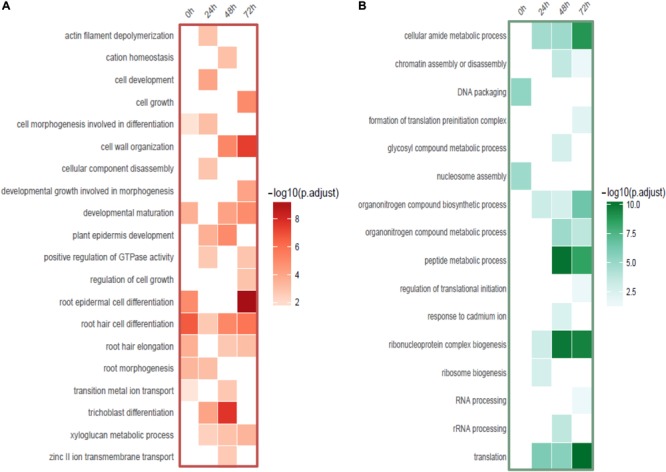
Functional categories of DE-genes detected from root hairs compared to non-hair epidermal cells. **(A)** Functional enrichment of up-regulated genes (red). **(B)** Functional enrichment of down-regulated genes (green).

Interestingly, a cell type-specific gene expression pattern was identified that was not related to the length of time of Cd stress (Figure 4A). We found hundreds of differentially expressed genes from root hair cells when compared to WRT and non-hair epidermal cells (Figure 4B; *padj* < 0.01). Gene ontology analysis was carried out to identify the functions of differentially expressed genes. In a comparison of root hairs and non-hair epidermal cells at the corresponding time point of Cd treatment, the genes governing root hair elongation, root hair cell differentiation, and cell wall organization were markedly up-regulated (Figure 5A). Under prolonged Cd treatment, increased expression of genes responsible for zinc(II) ion transmembrane transport, xyloglucan metabolic pathway, and developmental growth involved in morphogenesis were detected (Figure 5A). The Kyoto Encyclopedia of Genes and Genomes (KEGG) analysis revealed that the Cd stress-affected genes are associated with phenylpropanoid biosynthesis, oxidative phosphorylation, and phagosome function, suggesting a group of common regulators in this stress response. The PAT-Seq detected up-regulation of a group of root hair genes including *ACT2* (Actin 2, AT3G18780, log2FC = 3.12, *padj* = 0.01), ACT8 (AT1G49240, log2FC = 3.28, *padj* = 0.008) and *CSLD2* (cellulose synthase like D2, AT5G16910, log2FC = 5.2, *padj* = 0.008) from 5-day-old root hairs. Elevated expression was also found in xyloglucan endotransglucosylase/hydrolase (XTH) family, such as XTH12 (AT5G57530, log2FC = 5.16, *padj* = 0.001), XTH14, and XTH26 (AT4G28850, log2FC = 5.31, *padj* = 0.004), after 24 h of Cd stress. However, regulatory genes in translation and ncRNA processing were down-regulated under Cd stress. For example, we found reduced gene expression of eukaryotic translation initiation factor 3 family, including eIF3A (AT4G11420, log2FC = 4.3, *padj* = 7.4 × 10^-5^) after 24 h of Cd stress as well as eIF3B-2 (AT5G25780, log2FC = 5.97, *padj* = 0.003) and eIF3K (AT4G33250, log2FC = 4.9, *padj* = 0.009) after 72 h of Cd stress in root hairs. Also, a group of argonaute genes were down-regulated in root hairs after 72 h of Cd stress, such as AGO1 (AT1G48410, log2FC = 5.32, *padj* = 0.007) and AGO4 (AT2G27040, log2FC = 5.61, *padj* = 0.009), compared to non-hair root epidermal cells.

### Differentially Expressed PAC and Poly(A) Site Switching in Response to Cd Stress

**FIGURE 6:**
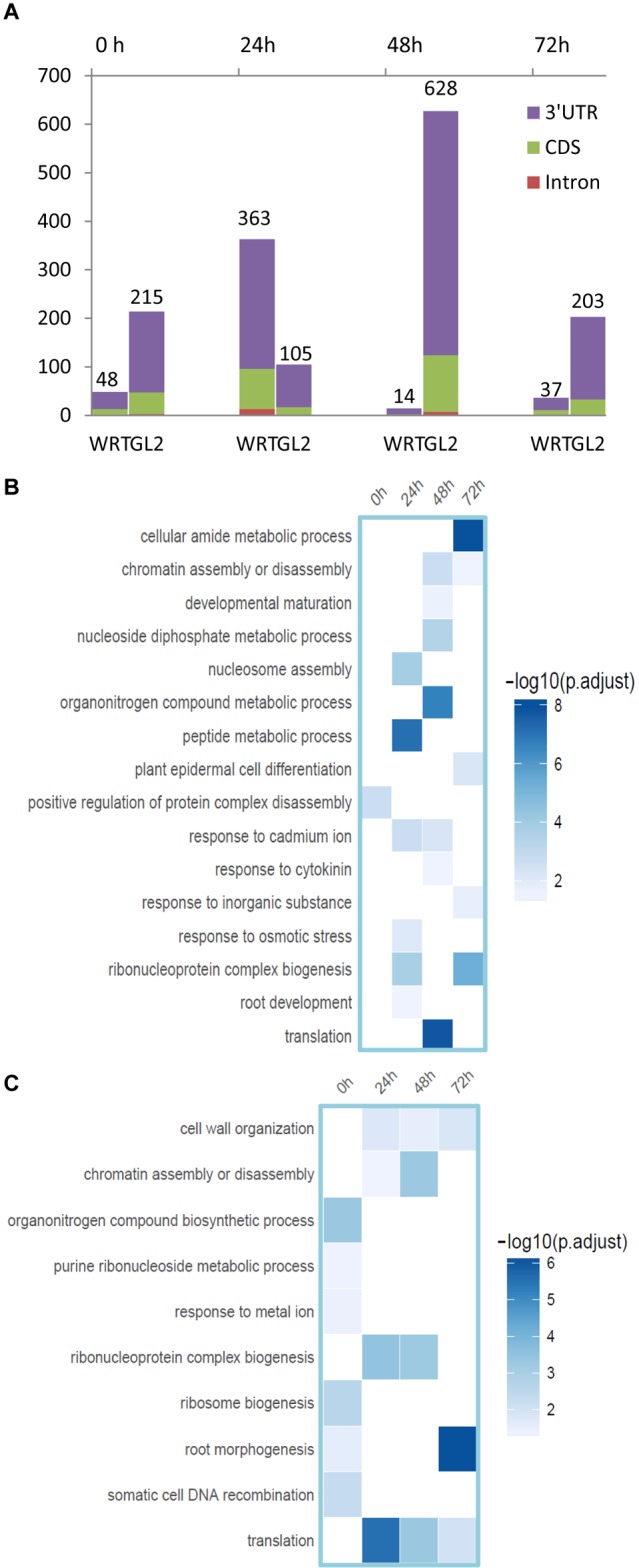
Identification of DE-gene, DE-PAC, and APA switch. **(A)** Number of root hair-specific DE-PACs identified in genic regions from the comparison with whole root control (WRT) and non-hair epidermal cells (GL2), respectively. **(B)** Functional enrichment analysis of DE-PAC identified from root hairs compared to non-hair epidermal cells. **(C)** Functional enrichment analysis of DE-PAC identified from root hairs compared to whole root tip control.

To assess if APA plays a role in the differentiation or function of different cell types against Cd toxicity, we then analyzed the APA profile from these datasets. A number of genes regulated by differentially expressed PACs (DE-PACs) were identified from a sample-to-sample comparison (Figure 6A and Supplementary Table S2). We explored the genomic regions of the DE-PACs. The highest number of root hair-specific DE-PACs was identified from 24 h Cd treatment when compared to the whole root control (Figure 6A). However, when compared to non-hair epidermal cells, the highest number of DE-PACs was found at 48 h Cd treatment (Figure 6A). A scarcity of DE-PACs was found in the 5′ UTR. Across all cell types and stress treatments, there were only three DE-PACs detected from 5′ UTR, two of them were identified from the comparison between root hair and non-hair cells at 0 and 48 h. One more 5′ UTR-derived DE-PAC was found by comparison between root hair cells and WRT at 72 h. The majority of DE-PACs were found in the 3′ UTR.

The majority of differentially expressed PACs were shown to be associated with genes with altered overall expression (Supplementary Figure S4), indicating APA may contribute to gene expression regulation. DE-PAC-affected genes detected from root hairs under Cd stress were enriched in plant epidermal cell differentiation and in root morphogenesis (Figure 6B,C), revealing that APA might be responsible for root hair defects under stress by quantitatively adjusting poly(A) site usage. We found DE-PAC usages from several genes related to root hair defects such as overexpressed PAC in the CDS region of *RHD2* (root hair defective 2, AT5G51060, log2FC = 6.18, *p* value = 0.003), and in 3′ UTR of *CSLD2* (AT5G16910, log2FC = 4.56, *p* value = 0.007) in root hairs compared to WRT after 72 h of Cd stress.

DE-PAC was also found to alter expression of genes playing functional roles in ribosome biogenesis, translation, and DNA recombination (Figure 6B,C), providing clues for the regulatory components in APA machinery. PAT-Seq detected specifically reduced PAC usage in root hair cells at 3′ UTR region of AGO4 (AT2G27040, log2FC = 4.64, *p* value = 0.007) and in CDS regions of several components in the family of eIF3 including eIF3A (AT4G11420) and eIF3G1 after 24 h of Cd stress.

The differentially expressed PACs also regulated genes responsive to Cd ion and osmotic stress. Under prolonged Cd stress, we found a reduced PAC usage in root hair cells compared to non-hair epidermal cells at CDS region in *GSH1* (glutathione synthetase 1, AT4G23100) after 24 h (log2FC = 5.03, *p* value = 0.005) and 72 h (log2FC = 5.33, *p* value = 0.003), and at 3′ UTR in *GDH1* (glutamate dehydrogenase, AT5G18170) after 48 h of Cd stress. ERD (early responsive to dehydration) is a family of osmotic stress-inducible genes in Arabidopsis (Akpinar et al., 2012). In a comparison of root hairs with non-hair epidermal cells after 72 h of Cd stress, reduced PAC usage at 3′ UTR of ERD6 (AT1G08930) was detected, whereas an increased PAC usage at CDS was found in ERD14 (AT1G76180, log2FC = 4.34, *p* value = 0.0001) was noticed. Specifically, we detected differential poly(A) site usage (*p* value = 0.092) and differential gene expression (*p* value = 0.006) of a transcription factor, UPBEAT1, in root hair cells at 24 h under Cd stress when compared to non-hair epidermal cells. UPBEAT1 was previously found to regulate the balance of cell proliferation and differentiation by alternating ROS signals (Tsukagoshi et al., 2010). Thus, our findings suggest a potential function of UPBEAT1 in root hair development under Cd stress through differential poly(A) site usage.

**FIGURE 7:**
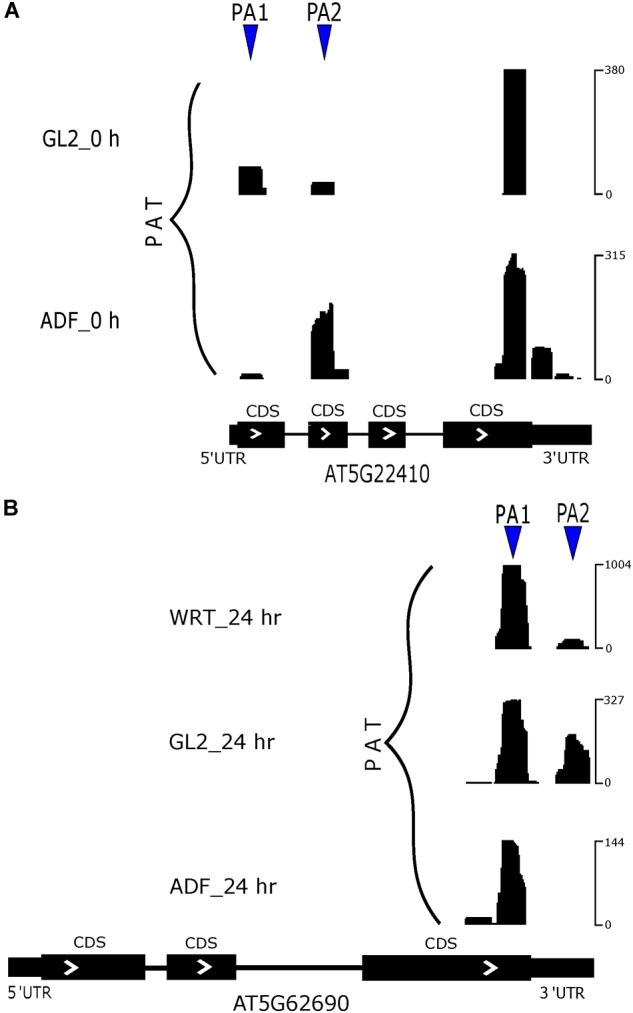
Poly(A) site switching in example genes. **(A)** APA switch of *RH18*. **(B)** APA switch of *TUB2* at 24 h of Cd stress.

There are only a small number of differentially expressed PACs that were regulated by poly(A) site switching (Supplementary Figure S3A). Differentially expressed genes regulated by both differentially expressed PACs and poly(A) site switching identified a group of root hair-specific genes (Supplementary Table S3). Among identified switching genes, a group of them were related to cell wall modification, which suggests a functional role of APA in root hair development. In a comparison between root hairs and non-hair epidermal cells from 5-day-old seedlings, *RH18* (*root hair specific 18*, AT5G22410) preferred to use the more proximal poly(A) site in non-hair epidermal cells, whereas usage of this preference was altered in root hairs (Figure 7A). Our PAT-Seq data also suggested one transcriptional factor, *SKIP* (SNW/SKI-interacting protein, AT1G77180) switched its preference on poly(A) site usage. Another group of poly(A) site switching genes were associated with various stress responses including Cd ion, cold, and oxidative stress, which indicates a common regulator of stress response. After 24 h of Cd stress, the preferred site was the proximal one in *TUB2* (tubulin beta-2/beta-3 chain, AT5G62690) from the root hairs compared to non-hair epidermal cells and WRT (Figure 7B).

## Discussion

### Cadmium Affects Arabidopsis Root Growth

As a non-essential toxic metal retained in soil, Cd has been suggested to affect many physiological and metabolic processes in root and leaf tissues. Cd was shown to inhibit root elongation in soybean in a concentration-dependent manner (Gzyl et al., 2015). However, limited work has been reported from the model organism *Arabidopsis thaliana*. In this study, we applied 100 μM CdCl_2_ to 5-day-old Arabidopsis seedlings and measured root elongation at 24, 48 and 72 h. As the control seedlings grew in a linear trend, Cd significantly inhibits the growth of root tips at 48 h (Figure 1B). Only subtle growth was detected at 72 h in Cd-stressed seedlings. Similar to the occurrence of root hair clusters at subapical region from oilseed rape seedlings (Sun and Guo, 2013), we also noticed the migration of root hairs toward root apex and clustered root hairs near the root tip. Consistently, the shift of the root hair cluster toward the root tip as a morphological defect in other stress responses such as copper exposure was observed to occur, and was due to a shift from cell proliferation to cell differentiation (Potters et al., 2007). Vacuole enlargement and altered microtubule patterning and cell wall organization have been shown to cause disruption of tip growth in root hair development (Galway et al., 1997; Sieberer et al., 2005; Gu and Nielsen, 2013). Collectively, toxic metal stress can significantly alter plant root development and root hair cell differentiation via altered ROS distribution and damaged cell wall organization.

### APA Modulates Oxidative Stress-Related Genes Under Cadmium Stress

Poly(A) tags were collected and pooled for analyzing single nucleotide composition of poly(A) signal, and we found an A+T-rich signal from 3′ UTR as well as an A+G-rich signal from coding region (Supplementary Figure S3). These data are similar to a previous poly(A) profiling from Arabidopsis leaf and seed tissues (Wu et al., 2011), revealing a consistent poly(A) signal profile from single cell types and whole tissue experiments. The collected PAC counts were combined from individual genes to identify differential gene expression. The majority of differentially expressed PACs were associated with genes whose expression also changed in response to the stress. However, only a few of these differentially expressed genes were identified as poly(A) site switching, indicating a distinct regulatory role of APA in a qualitative or quantitative manner. The highest number of DE-PACs was detected from root hair cells at 48 h Cd treatment when compared to non-hair epidermal cells. This suggests a higher resolution of poly(A) site profiling using individual cell types rather than whole organ in comparative analysis.

The KEGG analysis revealed the involvement of these up-regulated genes in phenylpropanoid biosynthesis and plant-pathogen interaction/detection pathways. Previous work in proteomic profiling of zinc-treated lettuce also found an enzyme in the phenylpropanoid biosynthesis pathway was up-regulated switching from flavonoids to flavonols and hydroxycinnamic acids synthesis (Lucini and Bernardo, 2015). Differentially expressed PACs were detected from gene function in the pentose and glucuronate interconversion pathways as well as the phenylpropanoid biosynthesis pathways, which were also enriched in chromium-responsive genes found in radish root (Xie et al., 2015). Moreover, increased expression of pentose and glucuronate interconversion genes was also revealed from lead-treated radish root (Wang et al., 2013).

Under prolonged Cd treatment, DE-PACs were also associated with antioxidant activity and peroxidase activity, which can be explained by the induced ROS generation under Cd stress (Chmielowska-Bąk et al., 2014). We found a differential PAC usage from the transcription factor UPBEAT1, which has been suggested to play a functional role in switching cell proliferation and differentiation under oxidative stress (Tsukagoshi et al., 2010). This finding proposed the involvement of stress-induced APA in post-transcriptional regulatory network.

### APA Affects Root Hair Gene Expression Under Cadmium Stress

Up-regulated genes identified from root hairs compared to non-hair epidermal cells were enriched in divalent metal ion transport and cellular response to extracellular stimuli, indicating that these groups of genes were responsible to the Cd-induced root hair defects, including increased root hair formation and shifted root hair clusters toward the root tip (Figure 5A). Our PAT-Seq data revealed DE-PACs in root hair defective genes including RHD2 and CSLD2. RHD2 is required for normal root hair elongation and knockout mutant of RHD2 showed large and spherical hair cells (Schiefelbein and Somerville, 1990). CSLD2 is required for cell wall organization in tip-growing cells, and mutation in CSLD2 resulted in root hair rupturing occurring in the later stage of root hair development (Bernal et al., 2008). These findings provide mechanistic insights into the alternative RNA processing of these root hair-defective genes, especially under abiotic stress. In agreement with a role of tubulin in cytoskeleton modification under ROS (Livanos et al., 2014), we detected poly(A) site switching in *TUB2* (Figure 7A). A previous study in soybean also found differential α- and β-tubulin isoforms under Cd stress (Gzyl et al., 2015). After 24 h of Cd stress, poly(A) switching was detected in several genes encoding cell wall proteins, *EXT10* (AT5G06640), *EXT12* (AT4G13390), and *ATXTH14* (AT4G25820). Elevated expression of actin genes, ACT2 and ACT8, was also detected; these genes are required for normal root hair tip growth (Kandasamy et al., 2009). We also found altered poly(A) site usage in *ATCSLE1* (AT1G55850) and *RH19* (root hair specific 19, AT5G67400) compared to non-hair epidermal cells after 48 h or 72 h of Cd stress. These findings indicate that APA might regulate a stress-responsive pathway common to heavy metals by mediating genes in cell wall modifications.

Other than these stress-responsive genes, APA is also involved in down-regulation of genes functional in translation initiation and ncRNA processing (Figure 5B). We found reduced gene expression in eIF3 factors, which was suggested to play a regulatory role under abiotic stress in plants (Echevarría-Zomeño et al., 2013). Components in RNA-induced silencing complex including AGO1 and AGO4 were down-regulated, implying a link between APA and pivotal regulators such as small ncRNAs (Wang et al., 2011).

### APA and Root Hair Differentiation

It is noteworthy that the PAT-Seq applied in the time course analysis of Cd stress in Arabidopsis seedlings revealed a cell type-specific gene expression patterns (Figure 4A). The set of up-regulated genes identified in root hairs compared to non-hair epidermal cells was enriched for those associated with root hair differentiation and root hair tip growth (Figure 5A). Moreover, a number of root hair-specific poly(A) site switching genes were found to be associated with biological processes including root hair growth and cell wall modification, suggesting a regulatory role of APA in finetuning gene expression in root cell fate specification. In comparison between root hairs and non-hair cells, we found poly(A) site switching in *RH18* (Figure 7B), which suggests a negative role in regulating root hair length (Won et al., 2010). APA switching events were detected in mRNAs encoding two transcription factors, *SKIP* and *RAP2.6* (AT1G43160). We also found altered usage of poly(A) sites in mRNAs encoded by *SCL8* (scarecrow-like 8, AT5G52510), which encodes a GRAS transcription factor specifically expressed in trichomes (Morohashi and Grotewold, 2009).

**FIGURE 8:**
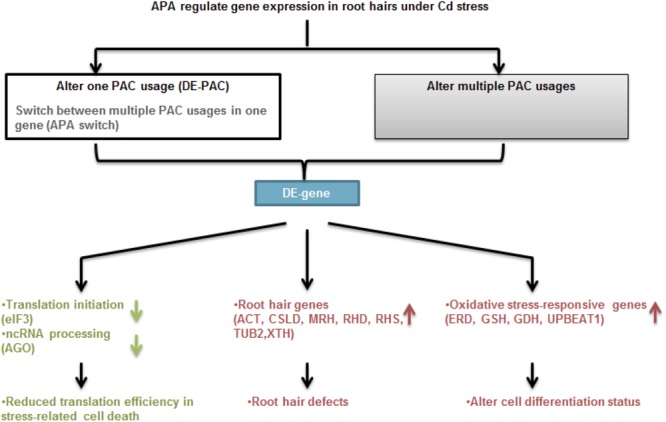
Proposed model for cell type-specific APA and gene expression under Cd stress. APA modulates gene expression through changing single PAC usage including switching poly(A) site preferences or majorly by altering multiple PAC usages in one gene. Under Cd stress, APA is involved in changing expression of genes function in translation initiation and ncRNA processing, as well as root hair defects and oxidative stress response, which are responsible for reduced translation efficiency, root hair morphology and unbalanced cell differentiation, respectively.

Taken together (Figure 8), FACS and PAT-Seq were used to profile poly(A) site usage in the Arabidopsis root epidermis. Intriguingly, we found a root hair-specific gene expression pattern in plants subjected to Cd stress. Poly(A) site switching was seen in an interesting group of genes that function in root hair differentiation, cell wall modification, and stress responses. While the impact of transcriptional activity should not be ignored, it will be interesting to further confirm the role of APA in these root hair-specific genes.

## Author Contributions

QQL and JC conceived the study, JC and GH performed the wet lab experiments. JC and CY did data analysis. AGH and CD-S co-supervised the study. JC, QQL, AGH, and CD-S wrote the manuscript. All authors approved the final version of the manuscript.

## Conflict of Interest Statement

The authors declare that the research was conducted in the absence of any commercial or financial relationships that could be construed as a potential conflict of interest.

## Footnotes

1 https://eucgenie.org/venn
