# Supplementary material for: Root Hair Single Cell Type Specific Profiles of Gene Expression and Alternative Polyadenylation Under Cadmium Stress
Source: Front Plant Sci. 2019 May 10;10:589. doi: 10.3389/fpls.2019.00589 (PMC6523994; doi:10.3389/fpls.2019.00589)
Supplement: Supplementary file 1 [file Data_Sheet_1.PDF]

Supplemental materials

Cao et al., **Root Hair Single Cell Type Specific Alternative Polyadenylation under Cadmium Stress**

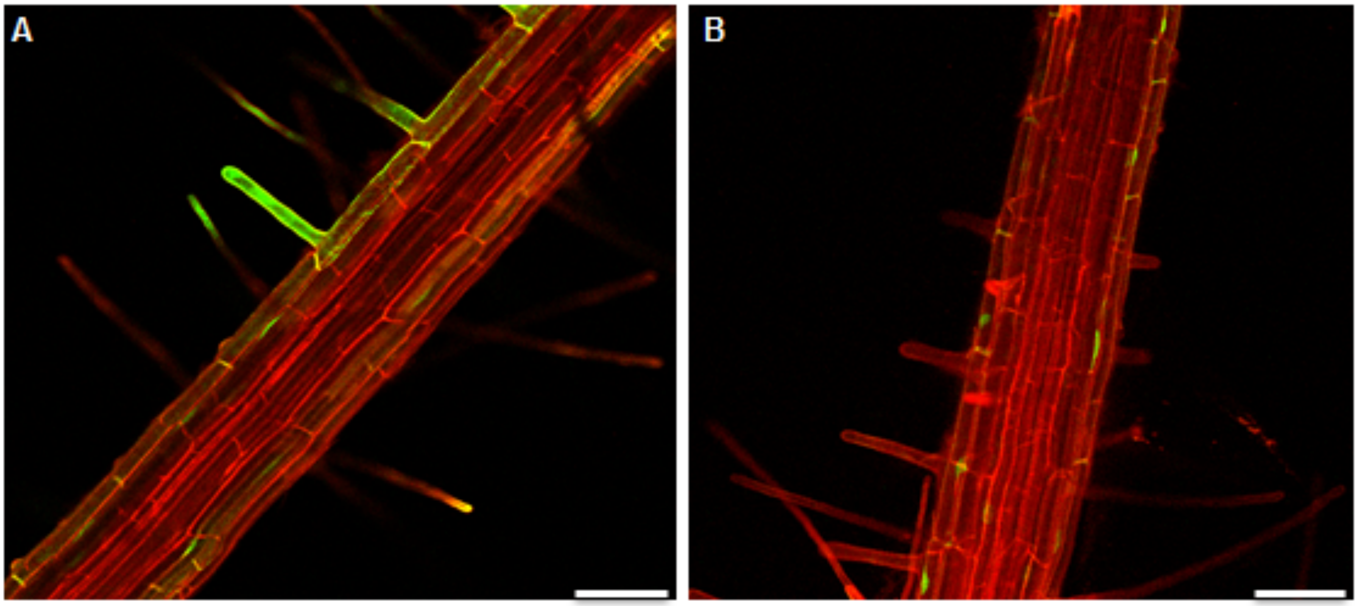

Supplementary Figure 1. Cell type-specific promoter driven GFP expression of INTACT lines applied in this study. (A) ADF8p:NTF/ACT2p:BirA, specific GFP expression detected in root hair cells. (B) GL2p:NTF/ACT2p:BirA, specific GFP expression detected in non-hair epidermal cells. Bar = 100 µm.

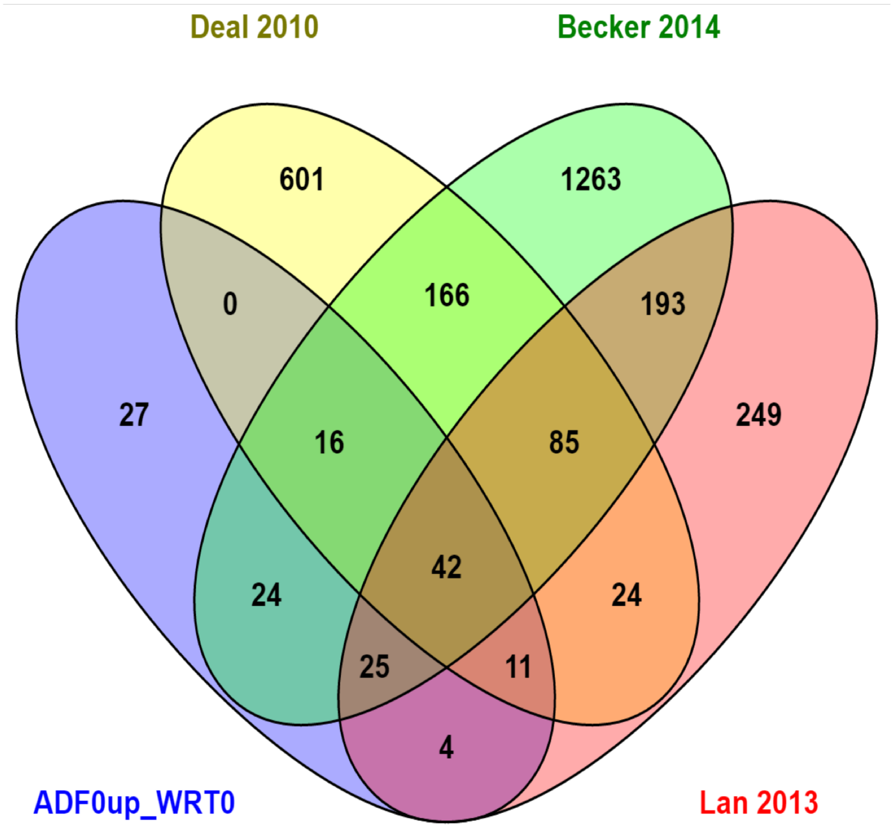

Supplementary Figure 2. Correlation analysis with published datasets. Venn diagram represents the overlap of up-regulated genes in root hair cells at 5-day-old detected from PAT-Seq (ADFup_WRT), root hair cells at 7-day-old by tilling array (Deal and Henikoff, 2010) and 5-day-old root hairs by microarray (Becker et al., 2014) and root hair cells at 5-day-old detected from RNA-Seq (Lan et al., 2013).

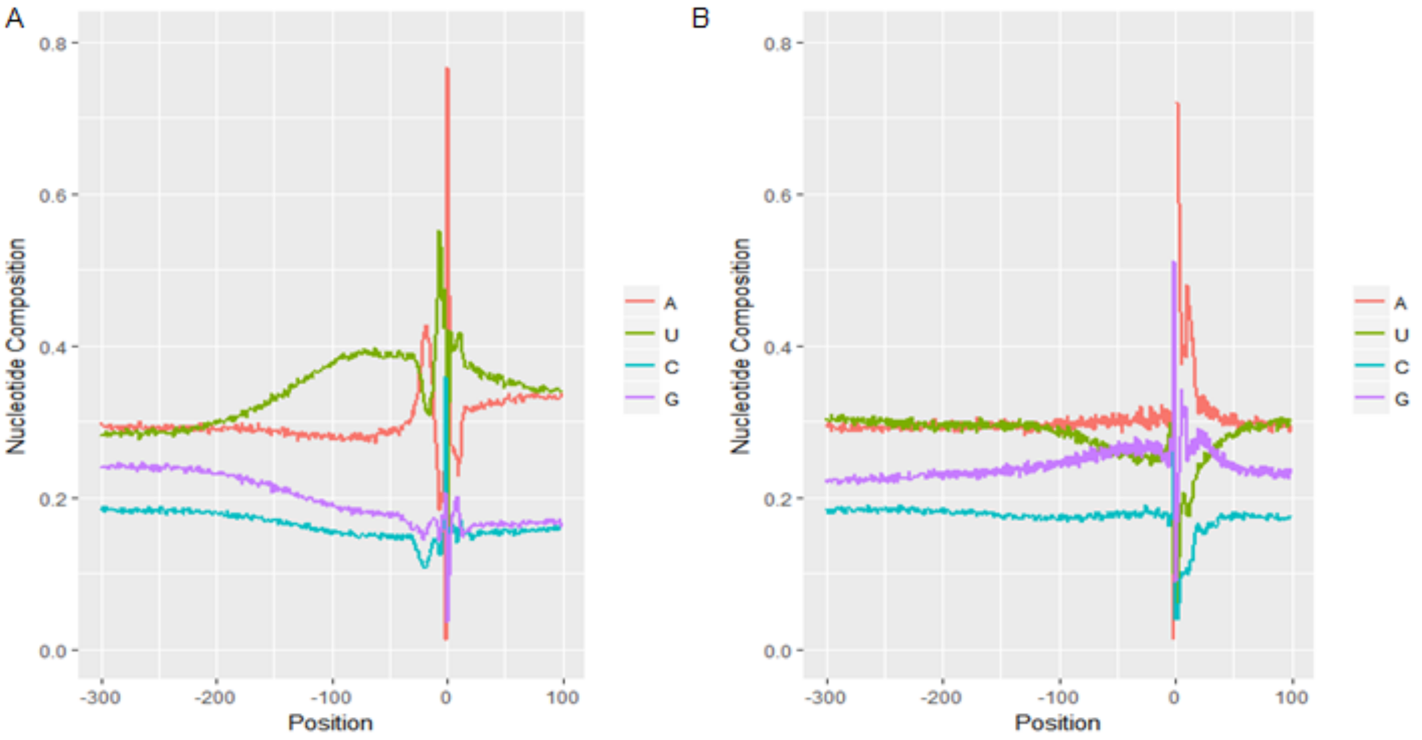

Supplementary Figure 3. Position-by-position analysis of poly(A) signal of PACs that mapped to 3' UTR (A), PACs that mapped to coding sequences (B). Y-axis, the proportions of different nucleotide at each position. X-axis, the flanking sequence (-300 nt to +100 nt) of PACs, and the position 0 represents the poly(A) sites.

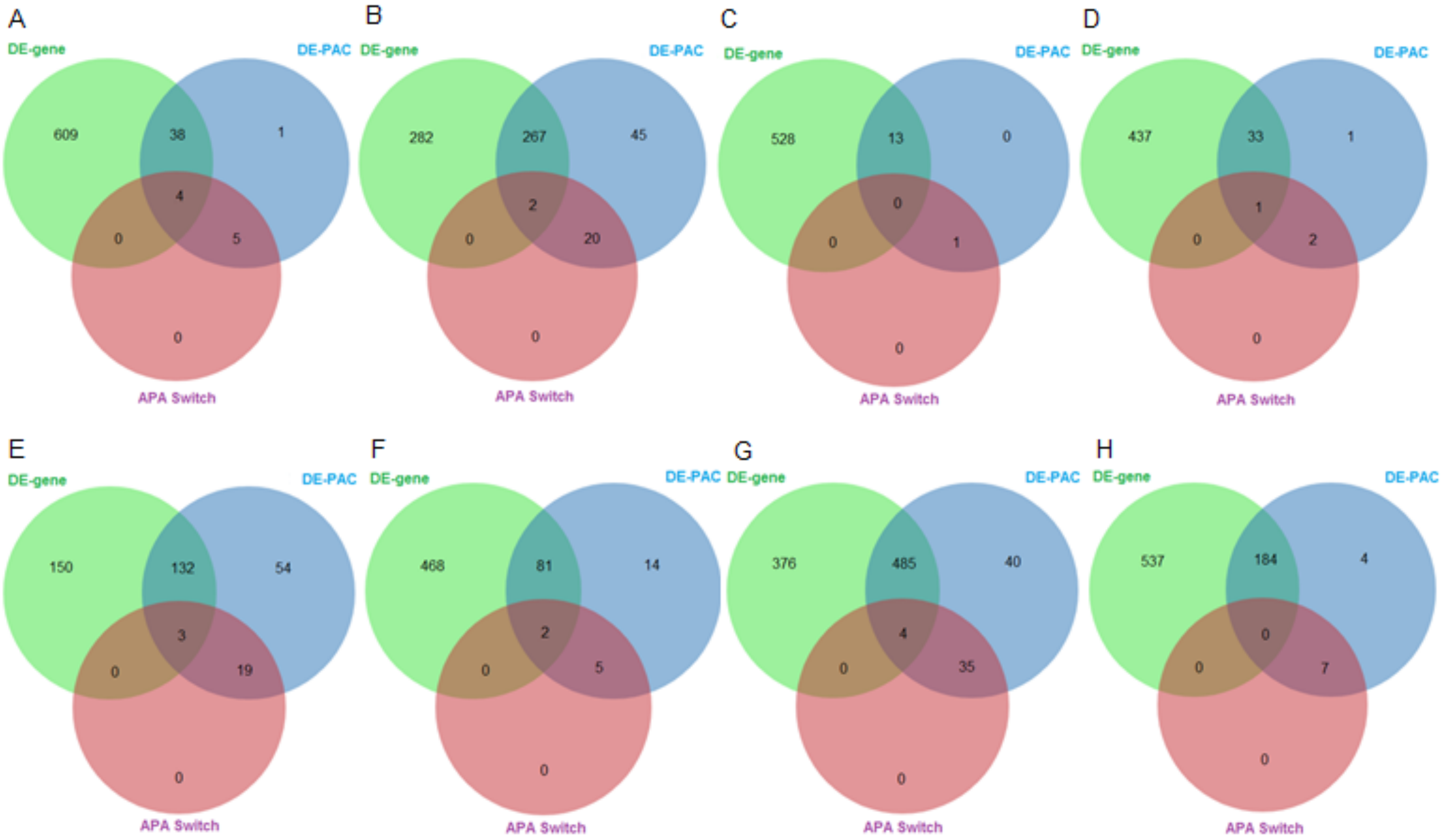

Supplementary Figure 4. Venn diagrams representing the overlapped genes detected by DE-gene (in green), DE-PAC (in blue), and poly(A) site switching (in pink). Top panel: Two-sample comparison between root hairs and whole root tips at 0 h (A), 24 h (B), 48 h (C), 72 h (D). Bottom panel: Two-sample comparison between root hairs and non-hair epidermal cells at time point at 0 h (E), 24 h (F), 48 h (G), 72 h (H).
