# Supplementary material for: Root Hair Single Cell Type Specific Profiles of Gene Expression and Alternative Polyadenylation Under Cadmium Stress
Source: Front Plant Sci. 2019 May 10;10:589. doi: 10.3389/fpls.2019.00589 (PMC6523994; doi:10.3389/fpls.2019.00589)
Supplement: Supplementary file 2 [file Data_Sheet_2.pdf]

Cao et al. Supplemental materials

Table S1. Primers used in PAT-Seq library construction

Primer name	Primer sequence
RT-PE series (bar-code is xxxxxx)	ACACTCTTTCCCTACACGACGCTCTTCCGATCTNNXXXXXXTTTTTTTTTTTTTTTTTTTVN
SMART 7.5	CGGTCTCGGCATTCCTGCTGAACCGCTCTTCCGATCTGG+G
PE-PCR 1	AATGATACGGCGACCACCGAGATCTACACTCTTTCCCTACACGACGCTCTTCCGATCT
PE-PCR 2	CAAGCAGAAGACGGCATACGAGATCGGTCTCGGCATTCCTGCTGAACCGCTCTTCCGATCT
P5	AATGATACGGCGACCACCGA
P7	CAAGCAGAAGACGGCATACGAGAT

Table S2. Alignment information of PAT-Seq libraries

Sample	Cell Type	Cd stress	Reads	PAT	PAS	PAC[a]			
						5' UTR	Intron	CDS	3' UTR
WRT	Whole root tip	0 h	9,980,789	1,798,415	44,877	1%	5%	38%	56%
			8,781,700	1,092,357	44,438				
			8,366,393	1,476,736	47,185				
ADF	root hair	0 h	10,287,027	1,516,278	35,404	1%	4%	29%	66%
			9,135,112	1,722,100	40,082				
			10,055,330	1,851,594	39,503				
GL2	non-hair epidermal cells	0 h	8,839,389	1,286,526	30,032	1%	5%	38%	56%
			11,367,330	1,615,610	81,196				
			22,332,196	3,955,617	10,108				
WRT	Whole root tip	24 h	11,015,138	1,572,913	51,507	1%	5%	33%	61%
			8,634,690	1,075,832	66,326				
			8,254,846	1,521,342	48,794				
ADF	root hair	24 h	7,245,465	1,126,295	38,990	2%	5%	32%	61%
			9,864,419	1,365,878	38,108				

			18,560,524	2,998,355	43,943				
			6,289,179	1,028,827	65,305				
GL2	non-hair epidermal cells	24 h	13,428,730	1,559,194	49,636	1%	5%	33%	61%
			5,500,864	807,546	16,783				
			12,879,422	1,350,261	36,913				
WRT	Whole root tip	48 h	12,397,597	2,552,228	106,432	1%	6%	42%	51%
			17,761,010	2,347,663	37,385				
			21,613,016	3,093,044	32,729				
ADF	root hair	48 h	7,653,705	1,177,809	40,895	2%	5%	36%	57%
			25,345,162	2,998,355	70,295				
			6,404,123	716,351	55,585				
GL2	non-hair epidermal cells	48 h	34,817,491	5,290,245	143,569	1%	5%	35%	59%
			10,911,672	1,663,680	68,652				
			10,637,701	1,239,683	28,662				
WRT	Whole root tip	72 h	10,385,496	1,491,611	80,303	1%	5%	35%	59%
			9,389,175	1,597,390	25,506				
			10,067,129	1,531,129	16,914				
ADF	root hair	72 h	7,719,622	1,126,544	30,700	1%	4%	31%	64%

			7,857,221	1,249,991	45,894				
GL2	non-hair epidermal cells	72 h	8,644,954	1,301,264	66,900	1%	5%	35%	59%
			7,035,624	917,724	64,423				
			9,546,141	1,647,269	121,262				

[a]PAC supported by less than 10 PATs or more than 1000 PATs were discarded to prevent from amplification artifacts. Percentage of identified PACs from specific genomic regions was listed.

Table S3. Functions of poly(A) site switching genes detected from sample-to-sample comparison

Gene ID	Name	Description	ADF vs GL2[a] (h)				ADF vs WRT[b] (h)			
			0	24	48	72	0	24	48	72
AT5G22410	RH18	Root hair specific 18	L							
AT1G34510		Peroxidase superfamily protein	L		L					
AT3G07490	AGD11	A member of ARF GAP domain.	L							
AT2G32240		early endosome antigen	S							
AT1G08340		Rho GTPase activating protein with PAK-box/P21-Rho-binding domain-containing protein	L							
AT1G43160	RAP2.6	Encodes a member of the ERF (ethylene response factor) subfamily B-4 of ERF/AP2 transcription factor family.	L							
AT5G52510	SCL8	SCARECROW-like 8	S							
AT3G58750	CSY2	Encodes a peroxisomal citrate synthase that is expressed throughout seedling and shoot development.	L							
AT1G77180	SKIP	Encodes a putative transcriptional factor.	L							
AT5G17910		Cardiomyopathy-associated protein	L							

AT5G27850	RPL18C	Ribosomal protein L18e/L15 superfamily protein	S							
AT1G20440	ATCOR 47	Belongs to the dehydrin protein family.	L							
AT5G49760		Leucine-rich repeat protein kinase family protein	L							
AT5G07080		HXXXD-type acyl-transferase family protein	L							
AT2G40950	ATBZIP 17	bZIP17 appears to regulate transcription as part of a salt and osmotic stress response.	S							
AT5G67480	ATBT4	BTB and TAZ domain protein	L	L						
AT5G19560	GEF10	Encodes a member of KPP-like gene family.	L							
AT1G20970		Calponin-like domain protein	S							
AT4G09000	GRF1	Encodes a 14-3-3 gene, designated GRF1 chi.	L							
AT4G20260	ATPCA P1	Encodes a Ca2+ and Cu2+ binding protein.	S							
AT1G22360	ATUGT 85A2	UDP-glucosyl transferase 85A2	L							
AT4G14320	API2	Zinc-binding ribosomal protein family protein		L						
AT3G23990	HP60	Mitochondrial chaperonin HP.		L						
AT3G09260	BGLU2 3	Encodes beta-glucosidase.		S						

AT4G37410	CYP81F4	A member of CYP81F		L				S		
AT1G43170	ARP1	Encodes a cytoplasmic ribosomal protein.		L						
AT5G54370		Late embryogenesis abundant protein-like protein		L	S					
AT1G56070	LOS1	Encodes a translation elongation factor 2-like protein.		L						
AT5G62690	TUB2	Encodes tubulin beta-2/beta-3 chain.		S				S		
AT5G61350	CAP1	Encodes a tonoplast-localized receptor-like kinase.		S						
AT1G26270		Phosphatidylinositol 3- and 4-kinase family protein		S						
AT3G21810		Zinc finger C-x8-C-x5-C-x3-H type family protein		S						
AT5G04970		Plant invertase/pectin methylesterase inhibitor superfamily		S				L		
AT3G46040	RPS15AD	Regulated by TCP20.			S					
AT4G27090		Ribosomal protein L14			S					
AT2G27530	PGY1	Encodes ribosomal protein L10Ap.			S					
AT1G55850	ATCSLE1	encodes a protein similar to cellulose synthase			S					
AT5G67400	RH19	root hair specific 19				S				

AT3G11120		Ribosomal protein L41 family				S				
AT1G50920	NOG1-1	Putative GTPase involved in HA - and ABA-mediated signaling pathways				L				
AT3G01790		Ribosomal protein L13 family protein				S				
AT5G08590	ASK2	Encodes a member of SNF1-related protein kinases.				L				
AT4G08400		Proline-rich extensin-like family protein					L			
AT3G60330	AHA7	H^+-ATPase 7					L			
AT5G42020	BIP	Luminal binding protein involved in polar nuclei fusion during proliferation of endosperm nuclei.					L			
AT5G06640	EXT10	Proline-rich extensin-like family protein					L			
AT2G25670		hypothetical protein					L			
AT3G16420	JAL30	The PBP1(PYK10-binding protein 1) assists the PYK10 (beta-glucosidase complex) in its activity and may act like a molecular chaperone that facilitates the correct polymerization of PYK10.					S			
AT4G08350	GTA02	Global transcription factor group A2						L		
AT1G67230	ATLINC1	Encodes a nuclear coiled-coil protein.						S		
AT3G53020	RPL24	Encodes ribosomal protein L24.						L		

AT4G25820	ATXTH 14	Encodes a xyloglucan endotransglycosylase.						L		
AT4G13390	EXT12	Proline-rich extensin-like family protein						L		
AT3G58840	PMD1	Encodes peroximal and mitochondrial division factor 1.						L		
AT2G47110	UBI6	Polyubiquitin gene						L		
AT5G54670	ATK3	Encodes a truncated KatC polypeptide.						S		
AT5G43580	UPI	Predicted to encode a PR (pathogenesis-related) peptide that belongs to the PR-6 proteinase inhibitor family.						S		
AT1G23750		Nucleic acid-binding, OB-fold-like protein						L		
AT5G27120		SAR DNA-binding protein						S		
AT1G65440	GTB1	Related to yeast Spt6 protein, which functions as part of a protein complex in transcription initiation and also plays a role in chomatin structure / assembly.						L		
AT1G75750	GASA1	GA-responsive GAST1 protein homolog regulated by BR and GA antagonistically						L		
AT5G64100		Peroxidase superfamily protein						L		
AT2G43090	IPMI	One of these genes encoding the small subunit of isopropylmalate isomerase.						L		
AT2G17350		beta-mannosyltransferase-like protein						S		
AT1G48300	DGAT3	Diacylglycerol acyltransferase							S	

AT1G62480		Vacuolar calcium-binding protein-like protein								L
AT2G01060		myb-like HTH transcriptional regulator family protein								L
AT5G02500	ATHP70-1	Encodes a member of heat shock protein 70 family.								S

[a] In comparison between GL2 and ADF, ADF used the longer or shorter isoform due to the poly(A) site switching.

[b] In

comparison between WRT and ADF, ADF used the longer or shorter isoform due to the poly(A) site switching.
